# Spatiotemporal evolution and mechanisms of tourism efficiency and its decomposition: Evidence from 63 counties in Zhejiang, China

**DOI:** 10.1371/journal.pone.0297522

**Published:** 2024-02-23

**Authors:** Dandan Gu, Dong Xu, Fenglong Yu, Bing Hou

**Affiliations:** School of Tourism and Culinary Science, Yangzhou University, YangZhou, Jiangsu, China; Hosei University: Hosei Daigaku, JAPAN

## Abstract

Although efficiency analysis could reflect the state and quality of tourism’s economic development, no research has been conducted investigating the spatiotemporal evolution and mechanisms of county-level tourism efficiency. We quantified tourism efficiency and its decomposition in 63 counties of Zhejiang, employing the bootstrap data envelopment analysis (DEA), hot spot analysis, and quantile regression to explore the spatiotemporal evolution and influencing factors of tourism efficiency, and examine its driving and constraining mechanisms. The results uncovered obvious upward trends in the tourism efficiency of Zhejiang’s counties, with the mean value increasing from 0.285 to 0.688. Compared with scale efficiency, the influence of technological efficiency on the growth of comprehensive efficiency increased remarkably. Significant differences were evident in the spatial distributions of the identified hot and cold spots of comprehensive efficiency, which were respectively distributed in northern and southern Zhejiang. The distributions of decomposition efficiency were found to be spatially dependent. The driving mechanism of tourism efficiency involve two driving influences and two constraining influences, including economy and resource driving, market and traffic driving, industry and traffic constraining, and market and industry constraining. The findings of this study contribute to understanding of tourism efficiency growth in regional destinations and provide insights for strategic policymaking in various counties of Zhejiang.

## Introduction

With rapid social and economic development, the tourism industry has had an increasingly significant role in advancing regional economic growth, industrial structure optimization, and spiritual and cultural development, particularly in Chinese counties [1–3]. Counties are the most stable units of public administrative division in China, and local county governments have been assuming an increasing number of crucial responsibilities for supporting local, serving rural areas, and promoting urban-–rural integration [4–6]. As small-scale spaces with multiple rural destinations, counties’ tourism industry has been engaged in ongoing and rapid development since the reform and opening up of China.

Counties provide strong support for implementing a series of major decisions and initiatives as a fundamental unit of the Chinese national economy. County-level economy refers to the sum of all economic and social resources within a county, which is of great significance to regional coordination and urban–rural integration. This study endeavors to accurately investigate and understand the law of county-level economies to promote the high-quality development of county-level economies and associated national strategies. The tourism economy has a leading role in the entire economic system of most counties in China [7–9]. Extensive research has been conducted regarding the county-level tourism economy, with rich findings that provide useful guidance and proposed measures for the development and planning of county economies and coordinated regional development [6, 10–12]. Overall, previous research has primarily focused on county-level tourism, development, examined the evolutionary trajectories and change mechanisms of different regional economic patterns, or explored the interactive mechanisms between county-level economies and tourism elements using relevant indicators and analyzed the potential of destination development [9, 13, 14]. With increasing inputs and outputs, tourism efficiency eventually becomes the core competitiveness of county tourism that leads regional economic development. Therefore, it is essential to deepen the research on county-level tourism efficiency [14, 15].

Tourism efficiency refers to the economic benefits obtained by a region following expenditure reflecting the intrinsic relationship between the inputs and outputs of tourism economic activities, and is primarily determined by scale and technical efficiency [15, 16]. The level of tourism development is strongly associated with tourism efficiency, and an imbalance in tourism efficiency can restrict sustainable tourism development. Scholars in existing research mainly measured the tourism efficiency of a certain region or unit to reflect the development status of its tourism industry or economy. Furthermore, targeted measures and policy recommendations were proposed to improve the efficiency and increase economic benefits [3, 5, 14–16]. Therefore, tourism efficiency is the core issue of tourism research [5, 8, 16–18]. Previous studies measuring tourism efficiency have primarily used DEA, stochastic frontier analysis, and slacks-based measure-DEA models, among other approaches [19–23]. Applying these methods, some scholars have explored the evolutionary characteristics, spatial network structure, and influencing factors of provincial level tourism efficiency, proposing valuable suggestions for advancing high-quality tourism development in China [23–26]. In addition, the evolution of regional tourism efficiency and its mechanisms have been analyzed by measuring tourism efficiency [27]. Previous research has improved the input–output index system for evaluating tourism efficiency and analyzed its spatial spillover effects [28]. Gómez-Vega et al. (2022) evaluated the tourism efficiency of 149 countries worldwide, analyzing external factors that may determine tourism efficiency [29]. By reviewing the existing literature on county-scale eco-efficiency, rural tourism impact and tourism industry specialization, research on the spatiotemporal pattern and evolutionary characteristics of tourism efficiency is of great value and should be further investigated to better amplify the socio-economic effects of county-scale tourism industry [30–32]. Overall, the majority of the previous studies on tourism efficiency that have focused on spatial distribution and impact paths involves tourism destinations of different scales, such as countries, provinces and cities [8, 16, 21–25], but lacks small-scale re-search at the county level, which is not conducive to constructing structural models at different scales to guide local micro development. Most research has only provided simple descriptions and measurement using econometric models, and knowledge regarding the driving factors and mechanisms of tourism efficiency and its decomposition is fundamentally lacking.

Based on the discussion above, this study measures the tourism efficiency and its decomposition for 63 counties in Zhejiang Province from 2005 to 2019, exploring the evolutionary spatiotemporal characteristics and influencing factors by applying an efficiency evaluation model, hot spot analysis and quantile regression. We then construct a driving mechanism framework for county-level tourism efficiency and its decomposition through multidimensional comparisons employing spatiotemporal visualization. This study introduces novel small-scale research on tourism efficiency by focusing on 63 counties in Zhejiang Province, supplements the research methods used for measuring tourism efficiency by adopting the Bootstrap-DEA and hot spot analysis. On the basis of measuring tourism efficiency and its decomposition respectively, it portrays their dynamic spatiotemporal patterns and characteristics, so as to better guidance the development of Zhejiang’s tourism industry. In addition, there is little existing literature that explains the spatiotemporal characteristics and driving mechanisms of tourism efficiency using spatial econometrics such as hot spot analysis and quantile regression. This study contributes to the research on tourism efficiency at the micro level and providing theoretical guidance for the sustainable, efficient, and healthy development of regional tourism.

## Materials and methods

### Efficiency evaluation model

DEA is the most commonly used nonparametric linear programming approach for evaluating the relative efficiency of decision making units (DMUs) by handling multiple inputs and multiple outputs [33, 34]. It is usually applied to analyze regional data, and there are two main models, constant scale and variable scale. The former assumes that the reward for scale is constant, and measures the comprehensive technical efficiency, and the latter measures pure technical efficiency with variable returns to scale. The key to using this method is to find appropriate input and output indicators, and the efficiency of input and output it calculates refers to "relative efficiency", and the real efficiency value will be less than the value by using DEA. And the DEA model selected in this study is based on the input orientation [34, 35]. Comprehensive efficiency can be decomposed into the product of pure technical efficiency and scale efficiency. Comprehensive efficiency is an integrated measure and evaluation of the resource allocation capacity of the decision making unit, the efficiency of resource utilization and other aspects of the capacity, while technical efficiency is the production efficiency of the enterprise on account of the influence of factors such as management and technology. Scale efficiency characterizes the impact of input growth on productivity changes, and can be used to determine whether each county unit is in the increasing or decreasing scale payoff range, so as to make corresponding adjustments to the scale of production, in order to bring the county unit to the optimal state of the scale of production.

In addition, CRS and VRS are two commonly used models in DEA. They are used to assess the technical efficiency of DMUs, which is to determine whether each DMU is efficient by comparing the inputs and outputs. The CRS model assumes that scale efficiency is constant, so the amount of inputs and outputs have a linear proportionality. The VRS model is more generalized in comparison with the CRS model, because it assumes that scale efficiency is variable. There is a nonlinear proportionality between input and output. CRS is generally used to analyze technical efficiency at a fixed scale, while VRS is used to measure changes in scale [34, 35]. Tourism is a multi-input and multi-output industry, where inputs tend to be more critical and outputs lag a bit behind. In order to reflect the overall changes in tourism efficiency in Zhejiang, we regarded each county as a DMU. In the DEA approach, efficiency is the objective function value of a multicriteria linear programming model. The DEA model is defined as follows:

$$\left\{ \begin{array}{l} \min(\theta -\varepsilon (e_{1}^{T}s^{-}+e_{2}^{T}s^{+}))\\ s.t.\sum_{j=1}^{N}x_{jg}\lambda_{j}+s^{-}=\theta x_{g}^{n}\;g=1,2,\cdots ,G\\ \sum_{j=1}^{N}y_{jh}\lambda_{j}+s^{+}=y_{h}^{n}\;h=1,2,\cdots ,H\\ \lambda \geq 0\;n=1,2,\cdots ,N \end{array}\right.$$ (1)

where *θ* represents comprehensive efficiency and ranges between 0 and 1. *ε* is non-Archimedean infinitesimal. *s*^−^ *a*nd *s*^+^ that are not less than 0 are slack and remain variables, respectively. *e*_1_^*T*^ and *e*_2_^*T*^ are *h*-dimensional and *n*-dimensional unit vectors, respectively. *x*_*jg*_ and *y*_*jh*_ (all positive) are the known input (g) and output (*h)* of the *j*th DMU. *λ*_*j*_ ≥0 represents the variable weights, and *N* is the number of all DMUs.

Despite the merits of using the DEA approach in parameter estimation, it may result in bias in sample evaluation due to the neglect of statistical testing [19, 34]. Accordingly, the bootstrap-DEA model was proposed to address this deficiency, with specific steps described in the previous studies [33, 36, 37]. Considering its advantages in resampling, we adopt this model to evaluate the county-level tourism efficiency in Zhejiang Province. The score measured by this model is the correction value of the relative efficiency of each county, which is between 0 and 1. An efficiency score of 1 indicates that the DMU’s efficiency is optimal and falls on the estimated production frontier. The comprehensive efficiency calculated by the model can be decomposed into scale and technical efficiency, wherein the latter is under the assumption of variable returns to scale. Comprehensive efficiency is the product of the decomposition of scale and technical efficiency. Scale efficiency characterizes the impact of input growth on productivity changes, which can determine increasing or decreasing returns to scale, indicating the corresponding strategic adjustments of production scale that can be made for the county to reach the optimal production scale [22, 33].

### Hot spot analysis

Hot spot analysis can clearly identify where the high and low-value aeras of certain geographic variables are spatially clustered, which are known as hot and cold spots [38, 39]. We use it to identify the specific geographical locations of hot and cold spots of tourism efficiency in Zhejiang. Accordingly, this method could be achieved by calculating the index of *G*_i_* proposed by Getis and Ord (1992), with the following formula [38, 39]:

$$G_{i}^{*}=\sum_{j=1}^{n}w_{ij}x_{j}/\sum_{j=1}^{n}x_{j}$$ (2)

where: *w*_*ij*_ is the spatial weight between county *i* and county *j*, *x*_*j*_ is the tourism efficiency value of county *j*, and n is the total number of counties in Zhejiang. To facilitate comparison, *G*_i_* is generally standardized using the following formula:

$$Z(G_{i}^{*})=(G_{i}^{*}-E(G_{i}^{*}))/\sqrt{Var(G_{i}^{*})}$$ (3)

where *E*(*G*_i_*) and *Var*(*G*_i_*) are the mathematical expectation and variance of *G*_i_*, respectively. Hot, subhot, subhot, sub-cold spot, and cold spots of tourism efficiency in Zhejiang Province can be identified according to the value of Z(Gi*) [38, 39].

### Quantile regression

Quantile regression is an estimation approach for examining the linear relationships between quantiles of independent variables and the dependent variable, which allows us to determine all the conditional distribution of the dependent variable based on certain independent variables. The result of Quantile regression is more efficient and robust [20, 40, 41].

Suppose the probability distribution of the dependent variable *Y* is as follows:

F(y)=Prob(Y≤y) (4)

where the quantile *τ* (0 < *τ* < 1) of the dependent variable *Y* is defined as the minimum value of *y* satisfying *F(y) ≥ τ;* that is, the quantile of *q(τ) =* inf {*y*: *F*(*y*) ≥*τ*} can be obtained by the objective function *q(τ)*, which is defined as follows:

$$q(\tau )=argmin_{\xi }\{\tau \int_{y>\xi }\left|y-\xi \right|dF(y)+(1-\tau )\int_{y<\xi }\left|y-\xi \right|dF(y)\}$$ (5)

$$=argmin_{\xi }\{\int \rho_{\tau }(y-\xi )dF(y)\}$$

where the function *argmin*_*ξ*_ {} represents the value of independent variable *ξ* when taking the minimum value of the function, and *ρτ(μ)* = *μ(τ − I(μ < 0))* is the check function, which is asymmetrically weighted according to the value of *μ* [20, 40, 41].

### Indicators and data

Producing accurate quantification of tourism efficiency and its decomposition largely depends on the appropriate selection of input and output indicators; however, no standard has been acknowledged for such indicators in tourism research. This study references some existing relevant literature to make our measuring results more accurate [4–6, 23]. Representative data reflecting the inputs and outputs of tourism industries in 63 counties of Zhejiang level are included in the efficiency evaluation model on the basis of the data availability.

For input indicators, capital, labor, and land are the most basic production elements in economics; however, due to the characteristics of the tourism industry, accurate data to directly reflect capital, labor, and land cannot be obtained, therefore, alternative data were selected referencing existing studies [5, 35, 42, 43]. From the perspective of tourism attraction, we use tourism service and resource elements (i.e., tourist accommodations and reception facilities) as alternatives to indicate capital input. These elements include (international and domestic) travel agencies (*Tag*), star-rated hotels (*Star*), and AAA and above scenic spots (*Sce*). We obtain the total scores of these elements based on their numbers and relevant standards [5, 35, 42, 43]. In addition, the number of employees in the tertiary industry (*Emp*) of each county proxies as the indicator of the number of employees in the tourism industry to represent the indicator of labor input [25, 44, 45]. For practical reasons, tourism land input was not included in the model due to a serious lack of statistics. For the output indicators, previous research has suggested that it is scientific and feasible to use the total tourism revenue (*Rev*) and the total number of tourist arrivals (*Tar*) to measure counties’ tourism output [4–6, 36]. Therefore, we use these two indicators as the outputs in our efficiency evaluation model. The descriptive statistics of the input and output variables are shown in Table 1.

**Table 1:** Descriptive statistics of the input and output variables.

Type	Variable	Abbreviation	Observation	Mean	Std.Dev	Min	Max
Input variables	Travel agencies	*Tag*	945	28	53.5	1	895
	Star-rated hotels	*Star*	945	19	23.2	2	126
	AAA and above scenic spots	*Sce*	945	4	15.3	0	87
	Number of employees in the tertiary sector	*Emp*	945	20.89	34.7	1.4	406.7
Output variables	Tourism revenue	*Rev*	945	84.12	178.9	0.4	4004.5
	Tourist arrivals	*Tar*	945	647.3	865.8	11.1	20813.7

Determining spatiotemporal patterns of tourism efficiency in the counties is a complex process that involves the interaction of multiple driving factors of tourism. Following previous literature [2, 3, 5–7], we use six factors to explore the driving mechanism of the spatiotemporal evolution of county tourism efficiency and its decomposition, including economic development level, tourism resource endowment, market size, urbanization level, traffic development level, and governmental macro regulation, which factors are specified as follows. Counties’ economic development level (*Pgdp*) is proxied by GDP per capita [5, 46]; The tourism resource endowment (*Res*) is represented by the total score obtained by summing the scores of AAA and above scenic spots in a county [5]; Market size (*Mar*) is determined referencing counties’ population density, reflecting the influence of domestic consumer market on tourism efficiency [5, 35, 47]; Traffic development level (*Tra*) is measured using road mileage to represent counties’ traffic conditions and accessibility. For most counties in Zhejiang, self-driving remains the most common choice for tourists’ arrival [5, 35, 48]; Governmental macro regulation (Gov) is measured by the proportion of the added value of the tertiary industry in the county’s GDP because China’s key tourism resources are primarily state-owned assets and local governments lead county-scale tourism development [5, 44].

Zhejiang Province is China’s most economically developed region with high tourism marketization. The province’s tourism economy is at the forefront of the entire country because most of its counties have actively implemented the strategies of tourism rejuvenation, cultural tourism integration, and rural tourism development. The county-level tourism industry has become an important foundation for advancing Zhejiang’s tourism economy [5]. It has become the only pilot province in China to promote common prosperity through cultural and tourism integration due to the government’s high emphasis on the development of tourism economy. In the annual list of the top 100 counties in China in terms of comprehensive strength of county-scale tourism, Zhejiang has been ranked No. 1 in the country for five years continuously [49, 50]. The development of rural tourism in Zhejiang is booming. For example, among the national key demonstration villages announced in 2023, a number of towns and villages in Zhejiang have been selected. In addition, only the tourism statistics of Zhejiang Province are focused on the county level nationwide, which can provide detailed data statistics for this study. The development process of its county-scale tourism is representative and informative, so we selected 63 counties in Zhejiang Province as samples for this study. The data for this study are obtained from the Zhejiang Statistical Yearbook, the Yearbook of Zhejiang Tourism, the Zhejiang Culture and Tourism Yearbook, the Zhejiang Tourism Overview, Zhejiang Tourism Statistics, the Zhejiang Tourism Development Report, and the Statistical Bulletins of National Economic and Social Development for each city and county from 2005 to 2020. We examined official cultural and tourism websites and the statistical departments of each city and county to supplement any missing data. Considering changes in administrative divisions, we merged and processed the data for some county-scale units (such as Linan and Fenghua) according to the division standards of county-level administrative units in 2019. Finally, a total of 63 county-scale units are obtained.

## Results

### Spatiotemporal differentiation of tourism efficiency

**Fig 1:**
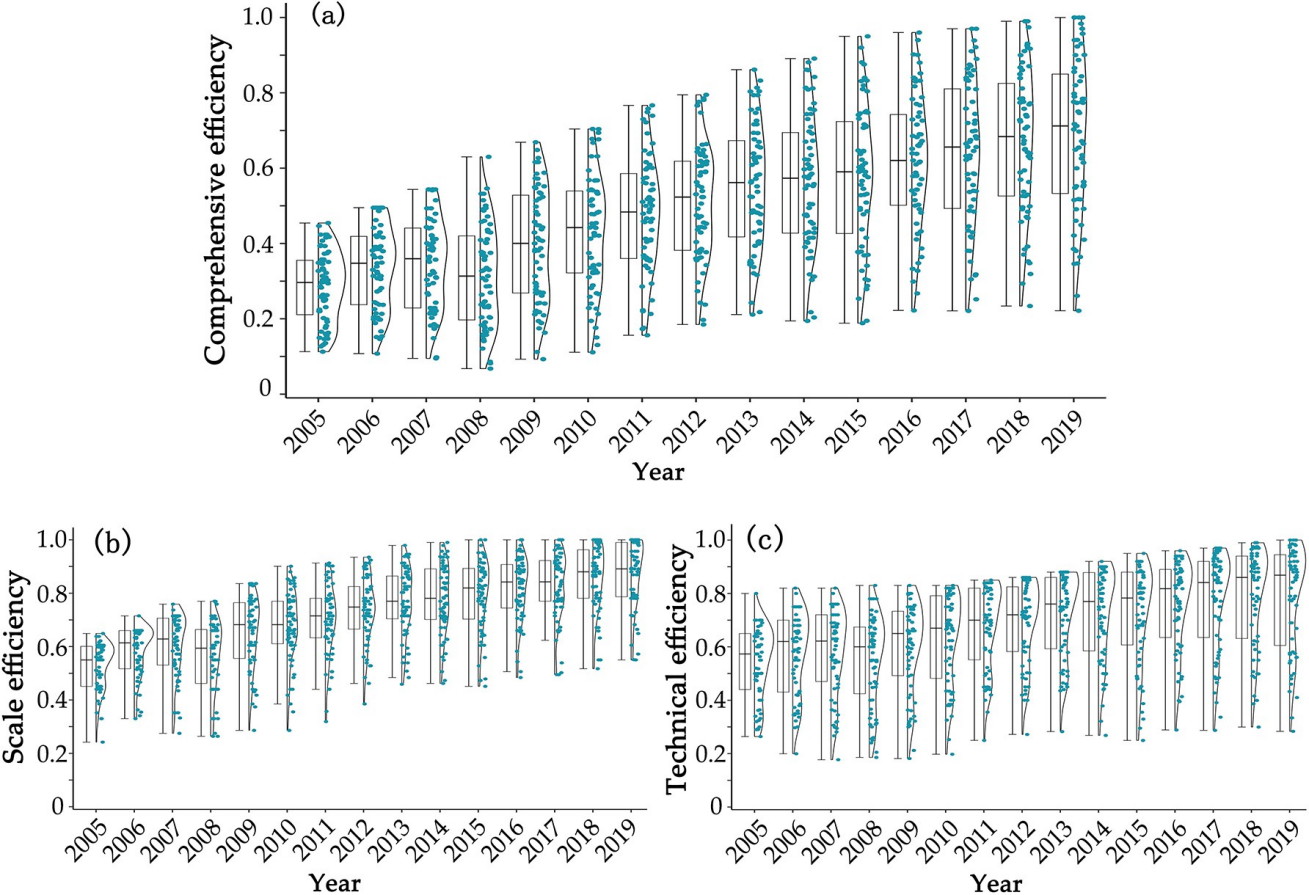
Tourism efficiency statistics and decomposition in Zhejiang Province’s 63 counties (2005–2019).

To analyze the overall changes in tourism efficiency and its decomposition efficiency more clearly, we present three boxplots (Fig 1) to illustrate the efficiency evaluation results of Zhejiang’s 63 counties, which are obtained using the bootstrap-DEA model. Comparing the maximum and minimum values of each year in Fig 1(A), reveals that the degree of comprehensive efficiency data dispersion in the 63 counties rose from 2005 to 2019 with less outliers, indicating that the differences in tourism efficiency between counties in Zhejiang are continuously widening. In addition, the mean value of comprehensive efficiency indicates an upward trend from 0.285 in 2005 to 0.688 in 2019, indicating significant improvement in tourism industry efficiency in the 63 counties. Examining Fig 1(B), the scale efficiency data of the 63 counties exhibits a low degree of discretization from 2005 to 2019, basically remaining at the same level each year. The scale efficiency scores mainly fall between 0.450 and 0.600 in 2005 and 0.710 and 0.957 in 2019. The mean value of scale efficiency during the study period was 0.716, increasing from 0.525 to 0.871 with an average annual growth rate of 3.68%. However, the technical efficiency data had a high degree of dispersion from Fig 1(C), indicating that regional differences in technological efficiency continuously expanded from 2005 to 2019; however, the boxplot continued to move upward over time, from a mean value of 0.539 in 2005 to 0.788 in 2019, demonstrating that the overall technical efficiency level in Zhejiang’s 63 counties continuously increased.

Based on efficiency evaluation results for Zhejiang’s 63 counties from 2005 to 2019, we analyse the spatiotemporal differentiation of tourism efficiency and its decomposition in 2005, 2012, and 2019. The high-value areas of comprehensive efficiency in Zhejiang show a trend of shifting from urban areas to surrounding counties. Specifically, counties with high comprehensive efficiency were primarily located in Jiaxing, Haining, Deqing, Taizhou, Shengzhou, Ningbo, and other surrounding areas in 2005, and the low-value areas were chiefly located in southern Zhejiang, which was likely limited by factors of regional economic development and location transportation. In 2012, although the comprehensive efficiencies of Hangzhou, Jiaxing, Ningbo, Huzhou, and Taizhou urban areas also increased, the counties’ growth rates were lower than those of the surrounding counties of Xiangshan, Tiantai, Panan, and Haining. The comprehensive efficiencies of low-value areas also improved slightly due to spillover effects from urban areas. By 2019, the values of comprehensive efficiencies of all counties in Zhejiang had significantly improved, with high-value areas primarily located in Xiangshan, Hangzhou, Huzhou, Jiashan, Anji, Jiaxing, Suichang, Yongkang, and Yiwu. The high- and low-value areas of comprehensive efficiency appear to be dispersed spatially. High-value areas are counties with high resource endowments and traffic accessibility, indicating that the focus of tourism development in Zhejiang has shifted from urban to rural areas.

The spatial distributions of scale and technical efficiency in 2005, 2012, and 2019 indicate that most of the counties in Zhejiang have transitioned to an efficiency-growth model that is dually oriented toward technology and scale. Specifically, the high-value areas of technological efficiency are mainly located in northern and eastern Zhejiang (with higher economic development) in 2005, such as urban areas of Hangzhou, Jiaxing, Deqing, Tonglu, Haining, Shengzhou, Cixi, and Ningbo and their surrounding counties; however, the low-value areas of scale efficiency are widely distributed, indicating that the early large-scale investments in Zhejiang generated serious resource waste. This also suggests that introducing advanced technology is crucial for improving the overall operational efficiency of tourism production and increasing tourism profits. In 2012, some counties in southwestern Zhejiang still had relatively low technological and scale efficiency, indicating that challenges such as unclear directions and paths of scale investment remained in some regions. A high degree of spatial coupling between the high-value areas of comprehensive efficiency and the high-value areas of technology efficiency and scale efficiency occurred in Zhejiang’s 63 counties in 2019. This took place in northern and eastern Zhejiang as well as central and western Zhejiang and some counties in southern Zhejiang such as Pingyang, Wenzhou, and Xianju. The scale and structure of tourism investments and science and technology inputs have had significant influence on promoting tourism development and overall tourism efficiency growth in these regions.

**Fig 2:**
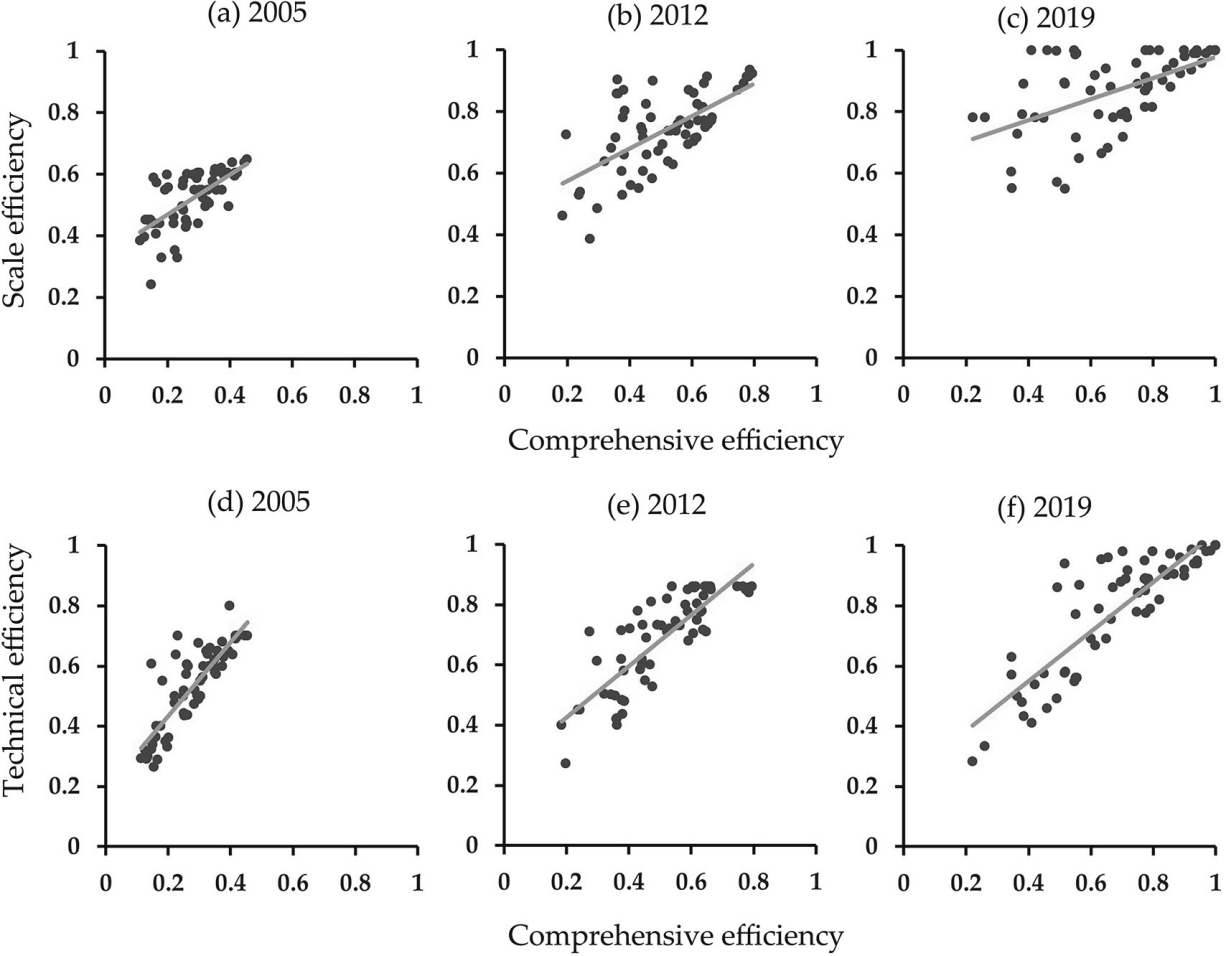
Corresponding relationship between tourism efficiency and its decomposition in Zhejiang Province’s 63 counties in 2005, 2012, and 2019.

To further examine the corresponding relationships between tourism efficiency and its decomposition in Zhejiang’s 63 counties, we present scatter plots with trend lines for 2005, 2012, and 2019 (Fig 2). The closer the trend line is to 45°, the greater the explanatory power of decomposition efficiency on comprehensive efficiency is. All the trend lines in Fig 2 deviated from the diagonal direction during the study period. The influence of scale efficiency on comprehensive efficiency gradually decreased, while the influence of technical efficiency significantly increased. The explanatory power of scale and technical efficiency on comprehensive efficiency were highest in 2005 and 2019, respectively. In addition, the scattered points exhibit remarkable extended characteristics with a trend from centralized to decentralized distribution in the longitudinal direction. This suggests that although both scale and technical efficiency increased, the former was higher than the latter in most counties, particularly in 2012 and 2019.

### Cold and hot spots of tourism efficiency

Hot spot analysis is used to examine the spatial agglomeration characteristics of tourism efficiency and its decomposition in Zhejiang’s 63 counties, and the results for 2005, 2012, and 2019 are illustrated in Table 2, revealing an increasing trend in the number of hot spots of the tourism comprehensive efficiency, while the number of cold spots decreased. Specifically, 3, 6, and 8 hot spots of comprehensive efficiency are identified in 2005, 2012, and 2019, accounting for 4.76%, 9.52%, and 12.70% of the 63 counties, respectively. hot spots were mainly distributed in Hangzhou, Deqing, Anji, and Huzhou in northern Zhejiang and Dongyang, Yongkang, and Panan in central Zhejiang. Cold spots of comprehensive efficiency were mainly located in Qingyuan, Ruian, Wencheng, Taishun, Pingyang, and Cangnan in southern Zhejiang, with 12, 7, and 7 cold spots in Zhejiang in 2005, 2012, and 2019, accounting for 19.05%, 11.11%, and 11.11% of the 63 counties, respectively. The scope of cold spots continued to decrease and move toward southern Zhejiang during the study period.

**Table 2:** Cold and hot spots of tourism efficiency and its decomposition in Zhejiang Province’s 63 counties (2005–2019).

Types	Year	Cold spots	Sub_cold spots	Hot spots	Sub_hot spots
Comprehensive efficiency	2005	Jinhua, Suichang, Longquan, Songyang, Yunhe, Lishui, Jingning, Qingyuan, Wencheng, Ruian, Pingyang, Cangnan	Taishun	Hangzhou, Haining, Tongxiang	Anji, Deqing, Huzhou
	2012	Jingning, Qingyuan,Wencheng,Ruian, Pingyang, Cangnan, Taishun	Cixi	Hangzhou, Tongxiang, Deqing, Panan, Yongkang, Dongyang	Changxing, Anji, Huzhou, Tiantai
	2019	Qingyuan, Wencheng, Taishun, Pingyang, Cangnan, Ruian, Cixi	Jingning	Hangzhou, Anji, Deqing, Huzhou, Dongyang, Panan, Tiantai, Yongkang	Jinyun
Scale efficiency	2005	Longquan, Qingyuan, Songyang, Yunhe, Jingning, Lishui, Taishun, Qingtian, Wencheng	Wuyi	Hangzhou, Deqing, Tongxiang, Jiaxing, Haiyan, Haining	Anji, Huzhou, Xinchang, Jiashan, Pinghu
	2012	Longquan, Yunhe, Jingning, Qingyuan, Taishun, Wencheng, Lishui, Cixi	Songyang, Qingtian, Ruian, Pingyang, Cangnan, Yuyao	Hangzhou, Deqing, Huzhou, Tongxiang, Jiaxing, Haiyan, Haining, Tonglu	Anji, Jiashan, Pinghu, Changxing, Zhuji, Shaoxing
	2019	Longquan, Yunhe, Jingning, Qingyuan, Taishun, Wencheng, Cixi		Anji, Hangzhou, Deqing, Huzhou, Tongxiang, Jiaxing, Jiashan, Pinghu, Tonglu	Zhuji, Shaoxing, Haining, Haiyan
Technical efficiency	2005	Jiangshan, Suichang, Jinhua, Longquan, Yunhe, Jingning, Taishun, Wencheng, Qingyuan, Songyang, Lishui, Jinyun, Xianju, Qingtian, Cixi, Yuyao	Ruian, Pingyang, Cangnan, Lanxi	Hangzhou	Anji, Deqing, Huzhou, Tongxiang
	2012	Yunhe, Ruian, Pingyang, Cangnan, Jingning, Wencheng, Qingyuan, Taishun	Longquan, Songyang, Lishui, Wenzhou, Qingtian	Hangzhou, Deqing, Huzhou, Dongyang, Zhuji	Anji, Changxing, Tongxiang, Haining, Yongkang,
	2019	Wencheng, Taishun, Ruian, Pingyang, Cangnan,	Jingning, Wenzhou, Qingtian	Hangzhou, Deqing, Huzhou, Jiashan, Yongkang, Suichang	Anji, Changxing, Tongxiang, Jiaxing, Zhuji, Panan, Dongyang, Yiwu, Pujiang

As is shown in Table 2, the hot spots of scale and technological efficiency have changed minimally, while cold spots have significantly decreased and moved southward. Specifically, the number of scale efficiency hot spots increased from six in 2005 to nine in 2019, while the spatial distribution changed slightly. The hot spots of scale efficiency were mainly located in northern Zhejiang from 2005 to 2019. Over the past 15 years, the number of cold spots of scale efficiency decreased from nine to seven in 2019, and was primarily located in Longquan, Jingning, Taishun, Yunhe, Qingyuan, and Wencheng in southern Zhejiang. However, the number of cold spots of technological efficiency decreased significantly from 15 (23.81%) in 2005 to 5 (7.94%) in 2019, and only located in Ruian, Wencheng, Taishun, Pingyang, and Cangnan. The number of hot spots of technical efficiency increased from one to six, with dispersed distribution. The hot spots of technical efficiency in 2019were primarily located in Hangzhou, Deqing, Huzhou, Jiashan, Yongkang, and Suichang. Overall, the scale and technological efficiency of the 63 counties improved and demonstrated significant spatial dependence with relatively regular changes in cold and hot spots. The spatial growth of tourism efficiency in most of Zhejiang’s counties from 2005 to 2019 may be obviously driven by technological investments.

### Influencing factors of tourism efficiency

This study adopts the quantile regression model to analyze the influencing factors of tourism efficiency, for which comprehensive efficiency (*CE*), scale efficiency (*SE*), and technological efficiency (*TE*) are used as dependent variables, respectively, and the six driving factors of *Pgdp*, *Res*, *M*ar, *Urb*, *Tra*, and *Gov* described above are used as independent variables. Additionally, a comparative analysis is conducted on the OLS and quantile regression results (see Table 3). Due to different quantile error terms, the influence of each driving factor on tourism efficiency presented differentiated trends and significance with changing quantiles. For CE, the influence of *Pgdp*, *Res* is significantly positive at low quantiles of 0.1~0.5, with regression coefficients of 0.011~0.054 and 0.004~0.006, respectively. The influence of *Mar*, *Tra* is significantly positive at quantiles of 0.7 and 0.9 but significantly negative at quantiles of 0.1 and 0.3. The influence of *Urb* is significantly negative at each quantile, indicating that it was an obstacle to county-level tourism in Zhejiang during the study period. The regression coefficients of *Gov* are negative but not significant at each quantile. For SE, the regression coefficients of *Pgdp* are not significant at each quantile, and the regression coefficients of Tra are significantly negative at low quantiles of 0.1~0.5. The regression coefficients of *Mar* are significantly positive at high quantiles of 0.5~0.9. The influence of *Gov* on SE is significantly negative at low quantiles of 0.1~0.5. The influence of *Res*, *Urb* is not significant. For technical efficiency, the regression coefficients of *Pgdp* are significantly positive, while those of *Urb* are significantly negative at each quantile. The influence of *Res* on technical efficiency is significantly positive at low quantiles of 0.1~0.5. The regression coefficients of *Mar* are significantly negative at low quantiles of 0.1~0.3. The influence of *Tra* is significantly negative at low quantiles of 0.1~0.3 but significantly positive at high quantiles of 0.5~0.9. The influence of *Gov* is not significant except at the 0.5 quantile.

**Table 3:** Estimation and test results of the quantile regression and OLS regression.

OLS/ Quantile Regression		ln*Pgdp*	ln*Res*	ln*Mar*	ln*Urb*	ln*Tra*	ln*Gov*	Constants
OLS	*CE*	0.022*	0.003*	0.016*	-0.018*	-0.057***	-0.102	0.900***
	*SE*	0.052*	-0.014	-0.010**	-0.003	0.064***	-0.029*	1.983**
	*TE*	0.068**	0.005	0.017*	-0.021**	0.101*	0.028	1.690*
0.1	*CE*	0.054**	0.004*	-0.056**	-0.021***	-0.058**	-0.048	0.258***
	*SE*	0.125	-0.006	-0.039	0.004	-0.077***	-0.079**	2.161**
	*TE*	0.152***	0.007**	-0.062**	-0.021***	-0.010*	-0.042	-0.321
0.3	*CE*	0.026**	0.004*	-0.005*	-0.014***	-0.045**	-0.013	0.053
	*SE*	0.073	-0.001	-0.014	-0.004	-0.069***	-0.029*	2.120**
	*TE*	0.118***	0.013***	-0.017*	-0.021***	-0.005*	0.026	-0.301
0.5	*CE*	0.011*	0.006*	0.005	-0.022***	0.021	-0.014	1.025***
	*SE*	0.068	-0.001	0.021*	-0.002	-0.043***	-0.016*	1.701***
	*TE*	0.101***	0.005*	-0.007	-0.028***	0.016*	0.108**	-0.122*
0.7	*CE*	0.020	-0.005	0.021***	-0.031***	0.024**	-0.011	1.010**
	*SE*	0.102	0.008	0.012**	-0.001	-0.020	0.025	1.202***
	*TE*	0.014	0.004	0.008	-0.027***	0.009*	0.007	0.890**
0.9	*CE*	0.015	-0.001	0.029*	-0.023***	0.046**	-0.009	1.276**
	*SE*	0.044	0.012	0.009*	-0.003	-0.020	0.048	1.130**
	*TE*	0.002	0.023	0.012	-0.002***	0.013**	0.106	0.925**

Note: *, ** and *** indicate significance at 10%, 5%, and 1% levels, respectively.

### Driving mechanism of tourism efficiency

**Fig 3:**
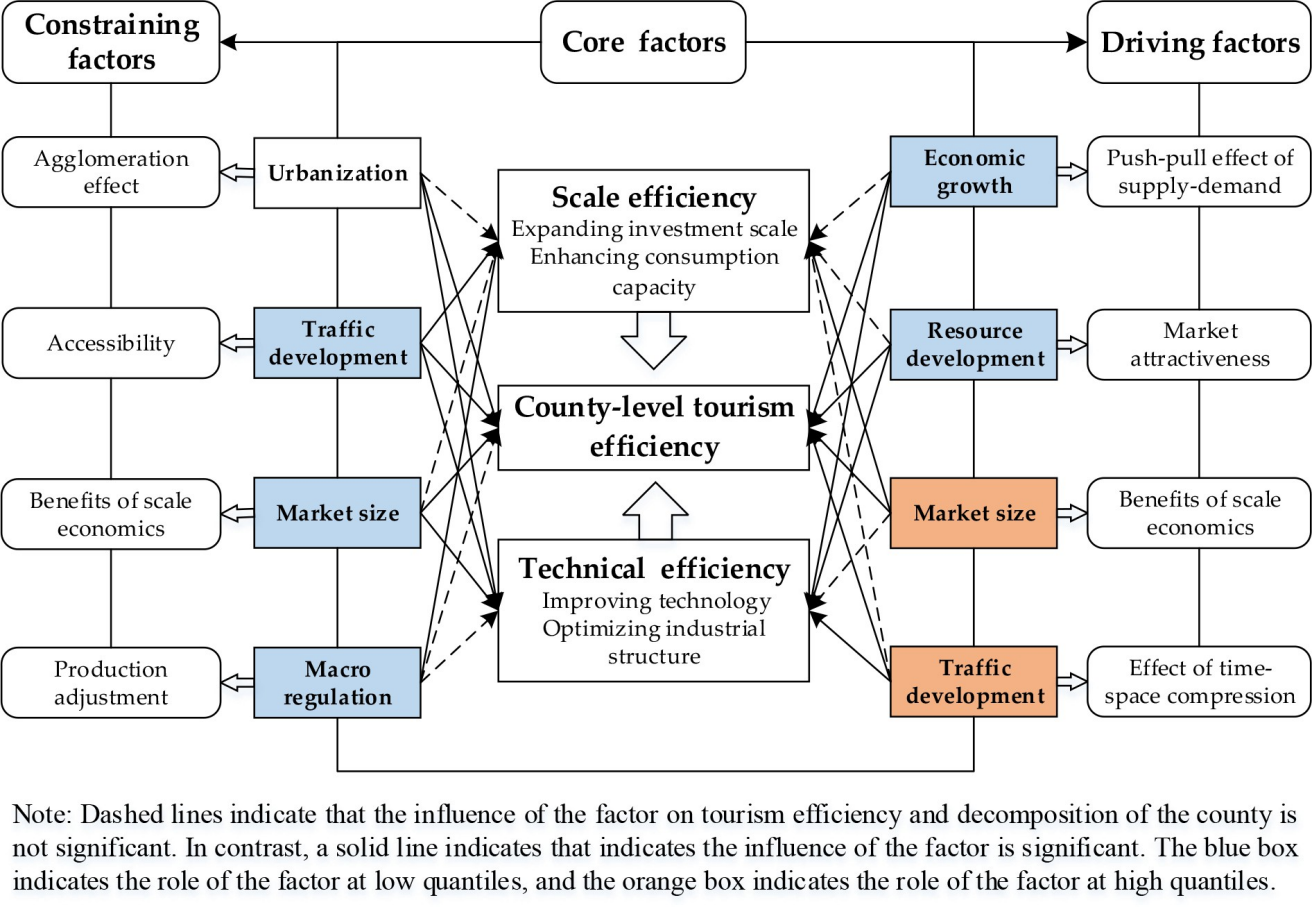
Driving and constraining mechanisms of tourism efficiency and its decomposition.

The positive and negative regression coefficients of the independent variables obtained though the quantile regression models at different quantiles of 0.1~0.5 and 0.5~0.9 are further divided into four types of high-quantile driving factors, low-quantile driving factors, high-quantile constraining factors, and low-quantile constraining factors. For example, high-quantile driving factors refer to independent variables with significantly positive coefficients at high quantiles of 0.5~0.9, which could promote the growth of tourism efficiency of Zhejiang’s counties. Low-quantile constraining factors refer to independent variables with significantly negative coefficients at low quantiles of 0.1~0.5, which may hinder the growth of tourism efficiency in Zhejiang’s counties. Based on the spatiotemporal evolutionary characteristics of tourism efficiency and its decomposition and the influencing factors analysis above, we construct a mechanism framework illustrating the tourism efficiency of Zhejiang’s 63 counties as shown in Fig 3, and divide the influencing modes into economy and resource driving type (Type I), market and traffic driving type (Type II), industry and traffic constraining type (Type III), and market and industry constraining type (Type IV). Specific explanations of the four refined types are as follows.

Type I: Economic development and resource endowments promoted tourism efficiency and its decomposition at low quantiles of 0.1~0.5 (Table 3). Economic development provided capital and material support for the tourism industry in the county and improved local residents’ tourism consumption capabilities. Counties with high resource endowments generally have stronger tourism market attractiveness, resulting in higher tourism efficiencies, indicating that scale investments in the early stages of tourism development may not have achieved the expected output. The promotional effects of economic development were primarily associated with increased investment in advanced technology and achieving information integration and product innovation. The role of resource endowments on SE is not significant. Using rich resources to establish innovative tourism products by improving TE in the early stages may be a wise approach for enhancing market competitiveness and tourism efficiency in Zhejiang’s counties.

Type II: Regional traffic development could promote the growth of tourism efficiency at high quantiles of 0.5~0.9. Destinations’ accessibility affects tourism operators’ management as well as tourists’ decisions. Market size could also promote the growth of tourism efficiency through improving SE at high quantiles of 0.5~0.9, indicating that expanding the regional market scale and improving the marketization level and market system can advance tourism development in a county. Additionally, traffic development also advances efficiency growth by improving TE at high quantiles of 0.7~0.9. With the development of information technology and accelerated transportation networking, tourism connections among counties have been strengthened to reduce travel time and distance costs, which is beneficial to the overall improvement of tourism efficiency in the later stage.

Type III: Urbanization and traffic development have certain inhibitory effects on tourism efficiency and its decomposition. Urbanization exerts a restraining effect primarily by constraining TE, but has no impact on SE at each quantile (Table 3). Urbanization level is reflected in the degree of spatial agglomeration of populations and industries. At present, the accumulation of tourism resources and the spatial agglomeration of the tourism industry facilitated by improved information technology do not seem to have generated tourism benefits for many counties. This is likely related to the values and lifestyles of urban residents. Compared to crowded tourist attractions, individuals who are seeking respite from their urban homes may tend to prefer undeveloped or underdeveloped scenic spots in surrounding counties. Traffic development has a negative impact, constraining both scale and technology efficiency at low quantiles, indicating that the understanding of tourists’ motivation and demands remains unclear. In the early stage of tourism development, scale and technology investments did not achieve the expected benefits and outputs.

Type IV: Market size and governmental macro regulation appears to have suppressed the growth of tourism efficiency and its decomposition to some extent. From the regression coefficients in Table 3, the market size of a county has an adverse impact on tourism CE by constraining the TE at low quantiles. Marketization of the tourism industry is an important force for promoting the tourism economy; however, technology inputs have not improved tourism efficiency, which is mainly attributable to the weak collaboration capabilities and division of labor in the early stage that partially led to low utilization efficiency of tourism production units. Although the impact of governmental macro regulation on CE was not significant from 2005 to 2019, it negatively influenced SE at low quantiles. This indicates that early industrial transformation by most county-level governments in Zhejiang did not seem successful and effective, and even resulted in a significant decline in the SE of the tourism industry.

## Conclusions and discussion

Research regarding the spatiotemporal patterns and driving mechanism of county-level tourism efficiency is essential for providing references for regional urban–rural integration, guiding local governments to reasonably manage resources to narrow regional disparities, and achieving high-quality tourism economy development [5, 24, 25]. As crucial components of China’s urban–rural system, counties are the links and bridges connecting cities and rural areas and cardinal public servants, leading urban–rural integration development and rural revitalization. Despite this critical micro level relevance, previous literature on tourism efficiency has focused more on provinces, cities, and certain key areas such as the national scenic areas and forest parks [2, 8, 9, 14, 25], neglecting the theoretical necessity and practical significance of conducting spatiotemporal county-level research. In addition, although previous research has explored the various determinants of tourism efficiency, no research has been conducted regarding the spatiotemporal relationships between tourism efficiency and its decomposition and more general mechanisms [8, 15, 17].

Considering the existing knowledge gaps above, this study evaluates tourism efficiency and its decomposition in Zhejiang Province’s 63 counties to measure the state of tourism economic development from 2005 to 2019 using the bootstrap-DEA model, examining the evolutionary spatiotemporal characteristics of comprehensive, scale, and technical tourism efficiency using GIS spatial analysis and hot spot analysis. Finally, we use a quantile regression model to explore the influencing factors and uncover the driving mechanism of tourism efficiency. Our study contributes to the tourism literature on efficiency analysis, industrial transformation, and urban–rural development. Meanwhile, this study revealed several interesting and relevant managerial insights for China’s culture and tourism sector and similar countries or regions. Accordingly, the main conclusions are threefold.

(1) Tourism efficiency and its decomposition in Zhejiang’s 63 counties showed a significant upward trend during the study period, with mean values of CE increasing from 0.285 to 0.688. The high-value areas of tourism efficiency presented a trend of shifting from core urban areas (such as Hangzhou, Jiaxing, Huzhou, Ningbo, and Taizhou) to surrounding counties (such as Xiangshan, Jiashan, Anji, Suichang, Yongkang, and Tiantai). China’s long-standing tradition of emphasizing urban tourism development has been moving toward smaller scale spatial units, which has occurred in the developed regions of Zhejiang. Moreover, the tourism efficiency growth of most counties has transformed from technology-oriented to technology- and scale-oriented trajectories. The impact of SE on CE has decreased, while the impact of technical efficiency increased markedly.
(2) In the past 15 years, the comprehensive tourism efficiency of the 63 counties studied exhibited overall change showing that the number of hot and cold spots increased and decreased, respectively. Significant differences are evident in the spatial distributions of the hot and cold spots. The hot spots of CE were mainly located in northern and central Zhejiang, while the cold spots were mainly located in southern Zhejiang. Additionally, although the hot spots of scale and TE of county-level tourism changed slightly, cold spots have significantly decreased and transitioned to southern Zhejiang (such as Taishun and Wencheng). From 2005 to 2019, the space–time distributions of Zhejiang’s cold and hot spots of tourism scale and TE exhibited significant spatial dependence.
(3) Due to obvious differences in resource endowments and economic foundations and significant geographical imbalance in tourism market, tertiary industry, and traffic conditions development in the later period, tourism efficiency and its decomposition in Zhejiang’s 63 counties presented differentiated scores and changes from 2005 to 2019. According to the estimated results of the influencing factors of economic development, tourism resource endowment, market size, urbanization level, traffic development, and governmental macro regulation using the quantile regression model, we examine the driving mechanism of tourism efficiency and its decomposition, dividing influences into the economy and resource driving type, the market and traffic driving type, the industry and traffic constraining type, and the market and industry constraining type. The study provides a theoretical and practical reference for tourism development and efficiency growth in the 63 counties of Zhejiang.

### Practical implications

This study makes outstanding contributions to the research on tourism efficiency analysis, with strong universal value for the evaluation of tourism efficiency, revealing spatiotemporal patterns, and identifying the influencing factors and related mechanisms. This study constructs a novel research method and applies spatiotemporal perspective regarding tourism efficiency and its decomposition, with three notable policy implications.

First, according to the overall changes in tourism efficiency and the spatial transfer of high-value and low-value areas of CE in Zhejiang from 2005 to 2019, more efforts should be made to avoid the phenomena of low resource utilization, waste, and idleness caused by large-scale investments in county-level destinations in the early stages of tourism development. The spatial distribution of scale and TE truly capture the stages of the tourism economy and reflect the differentiated roles of tourism investment scale, industrial layout and technological support, providing useful references for clarifying the direction and paths of county-level tourism development. Prioritizing the interrelationship between tourism efficiency and its decomposition seems valuable for unifying tourism planning and implementing reasonable and feasible policies for tourism development that are tailored to local conditions [4, 5, 10].

Second, our cold and hot spots analysis of tourism efficiency and its decomposition demonstrates that each county should recognize the dual importance of scale layout and technology input in the process of tourism development. To achieve high-quality development and reduce regional differences in southern and northern Zhejiang, it is essential to grasp the spatiotemporal relationship between tourism efficiency and its decomposition efficiency and understand spatial dependence, strengthen the connections among core urban areas and surrounding counties, and promote tourism spillover effects from hot spots such as Hangzhou, Deqing, Anji, and Huzhou. Southern Zhejiang is host to many high-quality mountain and water resources. Effectively leveraging ecological advantages to improve tourism scale and TE is a key challenge that local governments such as Taishun and Wencheng must deeply consider [6].

Third, our findings on the influencing factors and driving mechanism show that understanding the influencing types of tourism efficiency and transforming constraining factors into driving factors can promote the overall growth of tourism efficiency and its decomposition in various counties. Notable differences are evident in the influence of different factors on the comprehensive, scale, and technical efficiency in Zhejiang’s 63 counties. It is crucial to pay attention to significant factors such as economic growth, market size, resource endowment, and traffic development to promote comprehensive and decomposition efficiency [3, 16, 24]. The four patterns of driving and constraining mechanisms of tourism efficiency and its decomposition in Zhejiang Province, can help different counties to accurately position themselves so that they can take individualized measures according to their own situation to promote the growth of tourism efficiency and the healthy development of tourism economy, such as attaching importance to the development of tourism resources and strengthening the marketing of related tourism brands. Overall, facilitating economic growth and superior resource development can significantly improve counties with low tourism efficiency, while optimizing traffic accessibility and enlarging market size can be effective for counties with high tourism efficiency.

### Limitations and future research

China’s tourism industry is gradually improving, and the driving economic impact of county-level tourism on urban–rural integration and rural revitalization is prominent. To transcend the macro context of previous research, it is of considerable theoretical and practical significance to explore tourism efficiency by conducting in-depth research on tourisms’ spatiotemporal evolution and driving mechanism at the multiple county level. Such studies can fill the gap in the research on smaller scale units of tourism efficiency in China and refine techniques for conducting further research on county-level tourism efficiency. Research findings can also help local governments clarify the current state of the tourism economy to strategically determine the directions and paths of future development and provide theoretical and evidence-based support for the rational allocation and economical use of resources [16, 23]. However, because the current concept of tourism efficiency is not yet unified, the measurement of tourism efficiency is limited to a relative category, and the selection of evaluation input–output indicators remains controversial. In addition, the available county-level data are limited in China, and relevant data are missing for Zhejiang Province from 2020 to 2022. Although this does not affect the results of the analysis, further efforts are needed to conduct more accurate evaluations of tourism development to provide more useful references for tourism industries in similar spatial units of China and similar countries and regions. This study takes 63 counties in Zhejiang Province as the research object to refine the driving mechanism of county-level tourism efficiency and its decomposition as a whole, but due to the limited space of the article, it does not put forward specific suggestions according to the actual situation of different counties, which will be analyzed in depth in the future research.
